# Breed-specific values for vertebral heart score (VHS), vertebral left atrial size (VLAS), and radiographic left atrial dimension (RLAD) in pugs without cardiac disease, and their relationship to Brachycephalic Obstructive Airway Syndrome (BOAS)

**DOI:** 10.1371/journal.pone.0274085

**Published:** 2022-09-02

**Authors:** Pia Saskia Wiegel, Rebekka Mach, Ingo Nolte, Fritjof Freise, Charanthorn Levicar, Kristina Merhof, Jan-Peter Bach

**Affiliations:** 1 Clinic for Small Animals, University of Veterinary Medicine Hannover, Foundation, Hannover, Germany; 2 Institute for Biometry, Epidemiology and Information Processing, University of Veterinary Medicine Hannover, Foundation, Hannover, Germany; UTAD: Universidade de Tras-os-Montes e Alto Douro, PORTUGAL

## Abstract

This prospective study aims to establish reference ranges for vertebral heart score (VHS), vertebral left atrial size (VLAS), and radiographic left atrial dimension (RLAD) in pugs. The impact of clinical severity of Brachycephalic Obstructive Airway Syndrome (BOAS), gender, body condition score, and body weight on VHS, VLAS, and RLAD were investigated. Intra- and interobserver correlation was determined. Correlation of radiographic scores to echocardiographic left atrial dimension was inspected. Additionally, for VLAS and RLAD, correlation to VHS was examined. Additionally, an assessment of thoracic and vertebral malformations was performed. Forty-seven privately owned pugs underwent physical examination, echocardiography, and thoracic radiography to determine cardiac health. Thirty-two pugs were eligible for establishing reference ranges for VHS in right lateral radiographs, which was 11.25 ± 0.62 (95% range, 10.1–12.8). Reference ranges for VHS in left lateral, and for VLAS and RLAD in right lateral radiograph were determined in 30 pugs. The VHS in left lateral radiograph was 11.01 ± 0.70 (95% range, 9.4–12.6), VLAS was 1.96 ± 0.38 (95% range, 1.1–2.8), and RLAD was 1.59 ± 0.34 (95% range, 0.7–2.4). Clinical severity of BOAS did not show any impact on radiographic measurements. For VLAS, a significant correlation to VHS was detected by all observers. No other variables had a consistent influence on the radiographic scores given by all observers. Interobserver agreement was almost perfect for VHS (0.89 on right lateral and 0.91 on left lateral image), moderate for VLAS (0.49), and fair for RLAD (0.22). More than one third of the entire study population (18 of 47 pugs) showed at least one thoracic cavity or spine abnormality, often leading to considerable changes in vertebral body shape and size.

## Introduction

Echocardiography is commonly performed to examine the heart and determine the left atrial size [1]. Estimating the chamber size via echocardiography is the clinical reference standard for detecting left atrial enlargement (LAE) [2]. Among others, the echocardiographic parameter, left-atrial-to-aortic-root diameter (LA:Ao), is the basis for staging patients with myxomatous mitral valve disease (MMVD) and determines the point of initiating therapy [3]. However, echocardiography is often not as readily available as radiography [4–6]. Therefore, thoracic radiography is used for estimation of LAE, as well [4]. For this purpose, quantitative radiographic scores for an objective assessment of cardiac and left atrial size have already been established: vertebral heart score (VHS), vertebral left atrial size (VLAS), and radiographic left atrial dimension (RLAD) [5, 7, 8]. Their diagnostic utility have been proven of detecting cardiac enlargement and LAE [2, 5, 6, 9–13].

Investigating distinctive features of breeds and establishing breed-specific reference values is important to further improve the applicability of radiographic scores [14]. In several studies investigating VHS, significant differences between breeds have been reported and breed-specific reference ranges developed, for example, in the Beagle dog, the Dachshund, the Australian Cattle Dog, and the Norwich Terrier [15–18].

Brachycephalic breeds like pugs show characteristic anatomical features: they have a compact body shape, barrel-chested conformation, and a relatively broad or rounded cardiac silhouette on radiographs, which can lead to false interpretation of cardiomegaly [19]. For VHS in pugs, a mean of 10.7 thoracic vertebral units (v) has been described [20], revealing a significantly greater mean VHS than the published references for various breeds [7]. Interbreed disparity for VHS has been continuously reported over the past 20 years and likewise, such differences were recently detected for VLAS and RLAD [21–23]. This demonstrates that proposing breed-specific reference ranges for the pug will benefit radiographic evaluation of the heart.

Brachycephalic dogs are often affected by Brachycephalic Obstructive Airway Syndrome (BOAS), which is a disease of the upper respiratory tract leading to a variety of clinical symptoms [24–27]. In this context, respiratory noises, increased inspiratory effort, stress, heat, and exercise intolerance, and in more severe cases respiratory distress (dyspnea), cyanosis or syncope may occur [24, 26–30]. Some of these mentioned symptoms overlap with those of dogs suffering from a cardiac condition [31]. Considering breed-specific occurrence of BOAS, investigation of pugs clinically affected and non-affected by BOAS and its impact on radiographic scores may further help to differentiate between pulmonary or cardiac disease in radiographs. Since the cardiac silhouette shows specific characteristics in brachycephalic dogs [19], it is possible that this is related to BOAS. Revealing the influence of BOAS severity on radiographic scores might help with interpreting measurements with regard to the clinical BOAS status, especially in cardiac patients. Furthermore, other variables like gender, body weight (BW) or body condition score (BCS) have shown to effect VHS measurements in individual breeds [14, 16, 20]. Therefore, influence of these variables on VHS, VLAS and RLAD in pugs was investigated in the present study as well.

Additionally, brachycephalic dogs show a high prevalence of thoracic cavity and spine malformations [32–35]. One previous study found a high occurrence of short vertebral bodies despite normal shape in brachycephalic “screw-tailed” dogs [33]. Normal shaped thoracic vertebrae are necessary for measuring the radiographic scores, because abnormal vertebral may alter the outcome [20]. Thus, a detailed inspection of the thoracic spine in pugs is mandatory in order to examine the applicability of the scores, since reference ranges for radiographic measurements are ultimately based on vertebral length.

In addition to breed-specific differences, variability between individual observers and different levels of expertise may cause considerable differences between VHS, VLAS, and RLAD measurements [19, 36, 37]. Thus, assessing agreement within and between different observers may highlight the practicability of the radiographic scores in pugs.

The aim of this prospective study was to establish values for radiographic scores VHS, VLAS, and RLAD in pugs after determining cardiac health via echocardiography and to inspect whether BOAS and other independent variables had an impact. Correlations to LA:Ao and VHS (for VLAS and RLAD) as well as intra- and interobserver agreement were assessed.

## Material and methods

### Animals and examinations

Forty-seven privately-owned pugs were prospectively examined at the Clinic for Small Animals at the University of Veterinary Medicine Hannover, Foundation, Hannover, Germany. All owners signed an informed consent in which they agreed to all examinations and possible future use of the acquired data. The responsible authority, the Lower Saxony State Office for Consumer Protection and Food Safety (LAVES), Germany approved the study (file no. 33.19-42502-05-19A424). Demographic data of all 47 pugs included in the study can be found in Table 1.

**Table 1:** Demographic data of all 47 pugs included in the study.

N = 47	mean/median	SD/IQR	range (min—max)
age* [years]	4.8	2.9–6.4	2–13
body weight [kg]	8.9	1.1	6.6–11.6
BCS	5.4	0.6	4–7
female (n/i)	24 (11/13)		
male (n/i)	23 (4/19)		

Abbreviations: BCS, body condition score; i, intact; IQR, interquartile range; kg, kilogram; n, neutered; N, number; SD, standard deviation.

Note: median and IQR are stated for non-normally distributed variables, marked with an asterisk (*).

To be eligible for the study, pugs had to be aged 24 months or older. After recording the case history, all dogs underwent a physical examination, blood sampling for complete blood count and biochemistry panel, echocardiography, thoracic radiography, assessment of thoracic cavity and spine malformations, and were introduced to an exercise test (ET). Assessment of BOAS was performed in a later stage of the study, with an established clinical, functional grading system utilizing an ET, modified to match the present study design [38, 39]. Dogs underwent a submaximal ET on a treadmill in an individual trotting pace. According to the results of the functional grading before and after the ET, dogs were allotted to clinically non-affected (BOAS-) and clinically affected (BOAS+) groups [38]. A detailed description thereof can be found in the supporting information (S2 Table). Patients were not sedated for any of the conducted examinations.

### Criteria for radiographic measurements and comparison with regard to BOAS

For establishing reference ranges for radiographic scores VHS, VLAS, and RLAD, only patients that did not show any signs of cardiac or other significant systemic disease (except for BOAS) in case history, blood laboratory test, physical examination, and echocardiography were included in further analysis. Dogs were excluded from radiographic measurements if they had thoracic spine malformations that considerably influenced radiographic scores, such as classifiable malformations of the thoracic spine (e.g. hemivertebrae, wedge-shape vertebrae) [33] and non-classifiable malformations of thoracic vertebrae, in which the vertebral body length notedly deviated from the remaining, physiologically shaped vertebral bodies. For the latter cases, the decision whether to include the dogs in the measurements was made by a board-certified radiologist (KM). Dogs were also excluded if they were poorly positioned, showed a distinct lack of cooperation during radiographic examination, or radiographic images were of a poor quality.

Investigations of the impact of clinical severity of BOAS were conducted with all patients that underwent the ET. If the dogs showed a lack of cooperation during the ET and a complete BOAS assessment could not be performed, these animals were excluded from a comparison of radiographic scores between BOAS- and BOAS+ pugs.

### Echocardiography

Transthoracic echocardiography with simultaneous electrocardiogram was performed by one experienced examiner (JPB) (Vivid E7 and E9, GE Healthcare GmbH, Solingen, Germany). Measurements were obtained in right and left lateral position following current guidelines and published methodology in veterinary literature [40–43]. In detail, visual assessment and measurement of standard-echocardiographic parameters in two-dimensional (2D-) Mode, M-Mode and Doppler Mode were carried out using a standardized protocol for each examination [44]. Only patients considered to be free of cardiac disease based on echocardiographic examination were included in the study, and their thoracic radiographs were used for radiographic measurements. In two-dimensional images, from a right parasternal short-axis view, left atrial diameter and aortic root diameter were measured and LA:Ao calculated in a conventional method as described in previous literature [40].

### Radiography

Dogs underwent radiographic examination of the thorax in right lateral and left lateral recumbency. For all radiographs, exposure in peak inspiration or as close as possible to peak inspiration was pursued. All thoracic radiographs were acquired using the same digital radiography system (Philips Bucky-Diagnost, Philips GmbH, Hamburg, Germany) and were analyzed using standard analysis programs with a DICOM viewer (easy image, VetZ GmbH, Isernhagen, Germany; OsiriX MD, Geneva, Switzerland).

Radiographic images were blinded, and measurements performed by a board-certified radiologist with more than seven years of experience in small animal clinical practice and veterinary diagnostic imaging (KM, observer 1), and two first-year small animal veterinary clinicians (PSW and RM, observers 2 and 3). Interobserver agreement was determined for all three observers. For determining intraobserver agreement, observer 2 additionally measured ten blinded, random images for a second time with a time interval of at least seven days.

### Radiographic measurements

#### Vertebral heart score (VHS)

**Fig 1:**
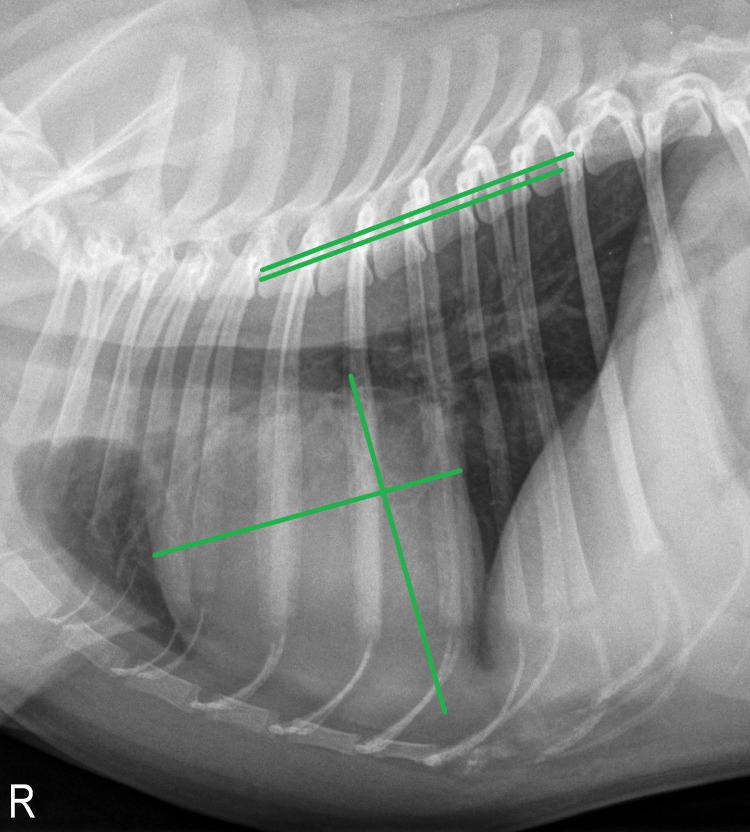
Right lateral thoracic radiograph of a two-year-old male pug. The green lines illustrate the measurement of the vertebral heart score (VHS), revealing a VHS of 11.4v in this subject.

The VHS was calculated as described previously [7], with slight modification: the short axis was placed perpendicular to the long axis so that its caudal edge terminated at the intersection point of the caudal cardiac silhouette with the ventral border of the vena cava [20]. The VHS was estimated by placing both lines of the short and long axis over the thoracic spine starting at the fourth thoracic vertebra (T4) and counting the vertebral bodies as units to the nearest 0.1v (Fig 1). One v was defined as the length of the vertebral body and its caudal disc [36]. VHS was obtained from right lateral (VHS RL) and, if available, from left lateral (VHS LL) radiographic view.

#### Vertebral left atrial size (VLAS)

**Fig 2:**
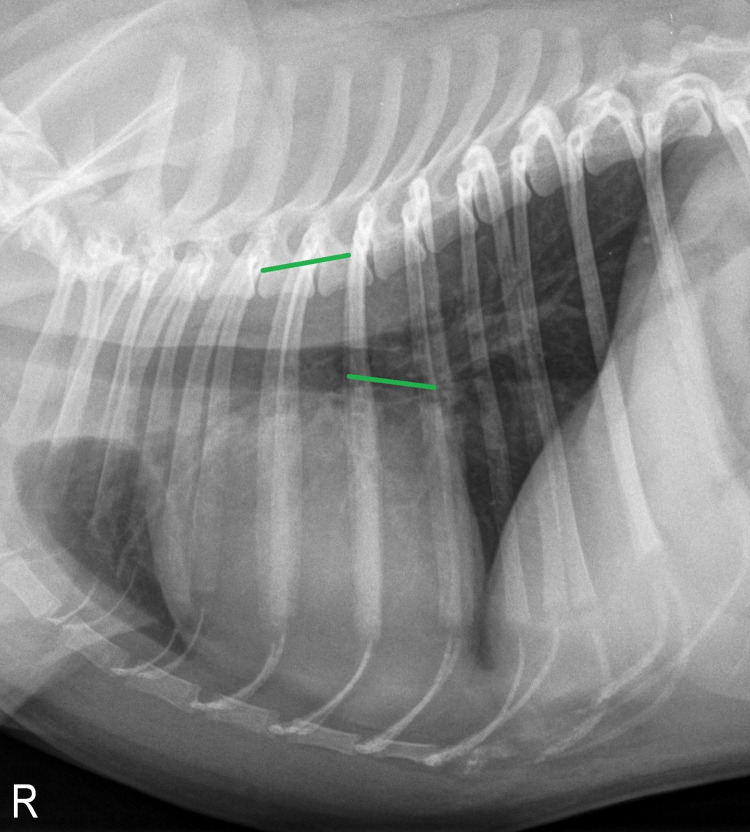
Right lateral thoracic radiograph of the same two-year-old male pug as in Fig 1, depicting the method for determining vertebral left atrial size (VLAS). In this case, VLAS turned out to be 1.6v.

The VLAS was obtained from right lateral radiographs, as described previously [5]: A line was drawn from the center of the most ventral aspect of the carina to the point of the most caudal aspect of the left atrium intersecting with the dorsal border of the caudal vena cava. A line of equal length was then positioned ventral and parallel to the vertebral column beginning from the cranial edge of T4. The VLAS was then assessed by estimating the length of the line placed over the thoracic spine to the nearest 0.1v (Fig 2).

#### Radiographic left atrial dimension (RLAD)

**Fig 3:**
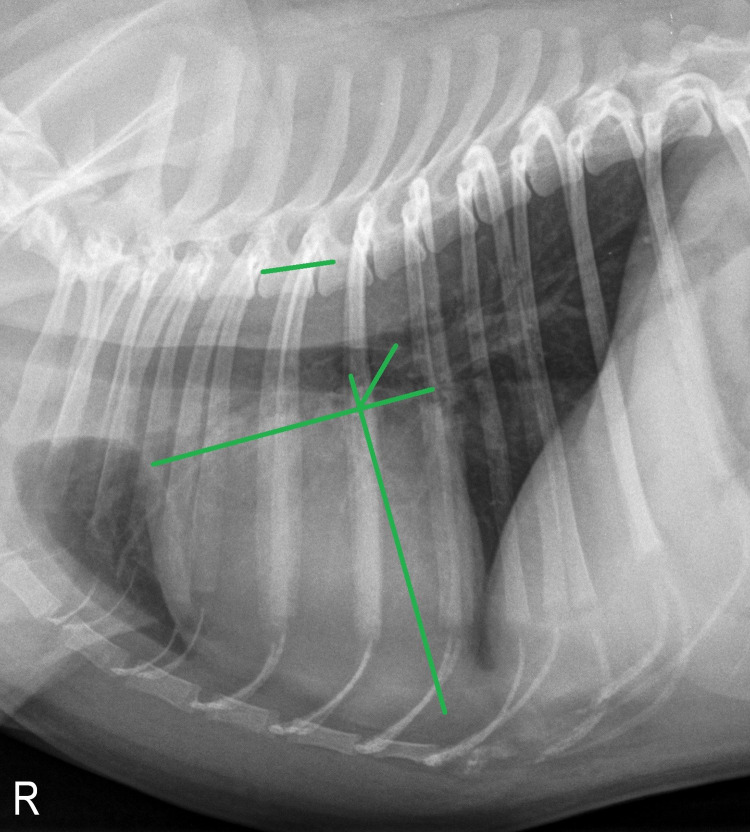
Measurement of radiographic left atrial dimension (RLAD) in a right lateral thoracic radiograph of the same two-year-old male pug as in Figs 1 and 2, being 1.3v in this subject.

The RLAD was obtained from right lateral radiographic images. For RLAD, VHS was applied with the caudal aspect of the short axis at the intersection point where the dorsal border of the vena cava caudalis meets the caudal border of the cardiac silhouette [8]. From the point of intersection of the VHS axes, a line bisecting the 90° angle was drawn to the dorsal edge of the atrium at a 45° angle. The length of this newly drawn line was then applied to the vertebral column, beginning at the cranial aspect of T4 and then estimated, as in VLAS, to the nearest 0.1v (Fig 3). In cases where the dorsal boundaries of the left atrium were difficult to assess (e.g., due to overlapping with neighboring pulmonary vessels), the most dorsal aspect of the soft tissue opacity seen at this level was used.

### Assessment of thoracic spine and sternal deformities

Thoracic vertebral malformations were classified according to a classification scheme for “screw-tailed” dog breeds [33]. Dogs that showed abnormal or irregular shapes of vertebrae, not allocable to the given classifications, were additionally recorded. Kyphosis and lordosis (abnormal dorsal and ventral spinal curvature) were qualitatively assessed on lateral images. It was distinguished between a general form, stretching across the thoracic spine, and a focal form, directly associated with a vertebral malformation. Pectus excavatum (PE, ventrodorsal deviation of the sternum) and pectus carinatum (PC, protrusion of the sternum) were assessed in laterolateral images [35]. The presence of any other types of thoracic, vertebral or sternal abnormalities were recorded.

### Statistical analyses

Statistical analysis was performed using commercially available software (SAS-Software, Version 9.4, SAS Institute Inc., Cary, NC, USA). All demographic variables as well as radiographic measurements were tested for normal distribution using the Shapiro-Wilk test and visual examination of histograms and Q-Q-plots. Descriptive statistics were reported as mean, median, standard deviation, and range.

To compare demographic parameters of age, BCS, and BW to BOAS groups (BOAS+/-) for each observer, the Wilcoxon rank-sum test was conducted. Differences with p < 0.05 were considered statistically significant.

### Reference values for VHS, VLAS and RLAD and comparison to previous studies

Reference values for VHS, VLAS, and RLAD were established for measurements of observer 1, creating a 95% prediction interval (reference interval, RI) using the samples quantiles (2.5% and 97.5% limits). For each lower and upper limit of RI, 90% confidence intervals (CI) were computed using the bootstrap method [45]. The VHS RL, and VHS LL were compared for all observers using the t-test. Either the t-test (if mean was specified) or sign test (if median was specified) was used to compare measurements of pugs of VLAS and RLAD with those stated in previous studies [5, 8, 21–23].

### Differences of radiographic scores in regard to BOAS and other independent variables, and correlation of VLAS and RLAD to LA:Ao and VHS

The Wilcoxon rank-sum test was conducted to compare each radiographic score between BOAS- and BOAS+ group for each observer. The t-test was used to compare weight, VHS, RLAD, VLAS of each observer between male and female dogs, and mean VHS measurements of dogs with low (1–5) and high (6–9) BCS. Pearson’s or Spearman’s correlation was used to determine correlation coefficients (r) between VHS, RLAD, VLAS, and age, BW, and LA:Ao and VHS RL (for RLAD and VLAS) for all observers.

### Differences between observers and intra- and interobserver agreement

To compare VHS, VLAS, and RLAD measurements between the observers, the Friedman test was used. To examine intra- and interobserver reliability, intraclass correlation coefficient (ICC) was determined. Estimates and their 95% confidence intervals were calculated based on a single rater, absolute-agreement, two-way random effects model. Intraclass correlation coefficient were interpreted as poor (0.01–0.20), fair (0.21–0.40), moderate (0.41–0.60), substantial (0.61–0.80), and almost perfect agreement (0.81–0.99) [46, 47].

## Results

**Fig 4:**
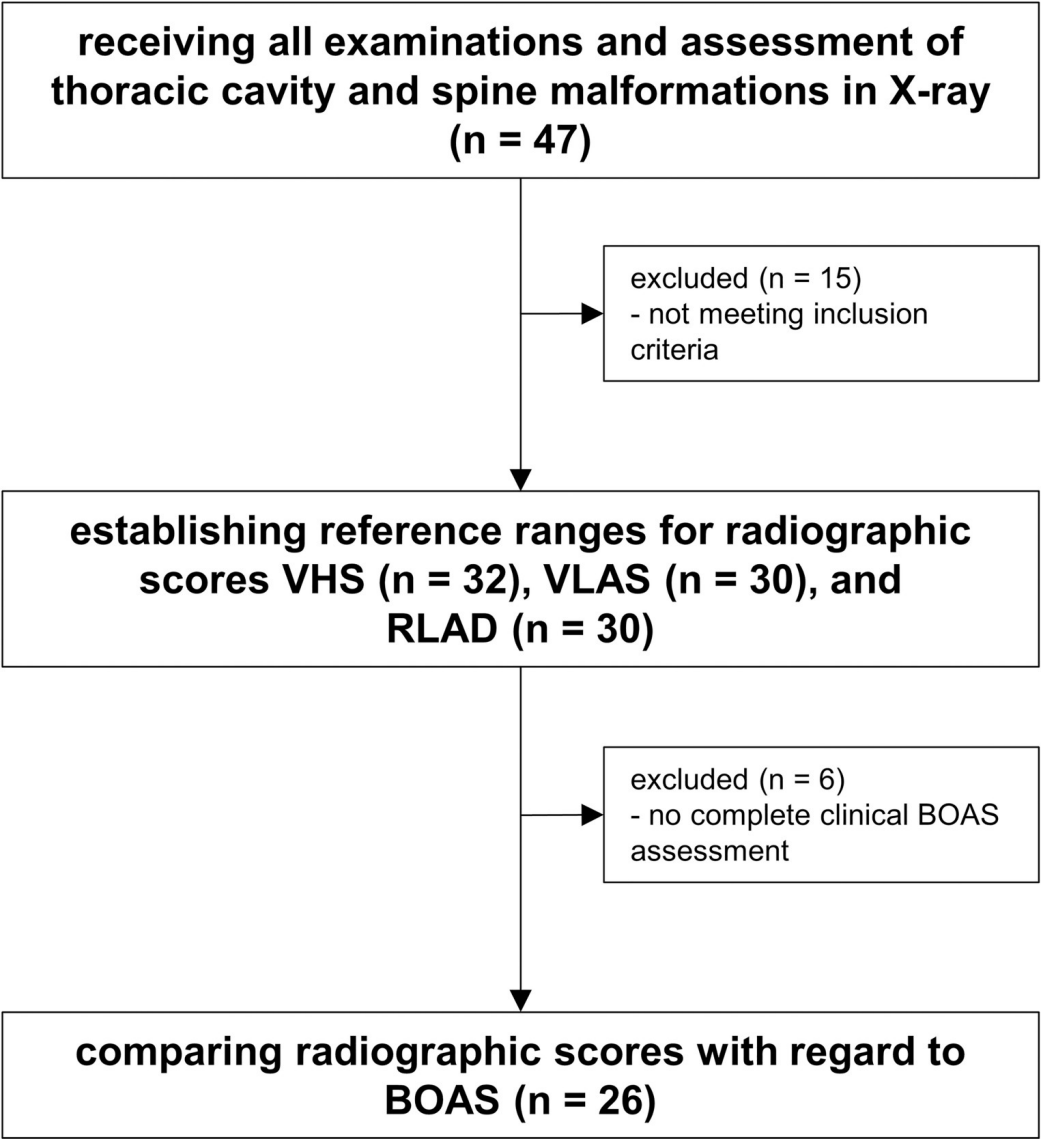
Assessment of eligibility.

Of 47 pugs, a total of 32 were eligible for radiographic measurements and were included in investigations regarding radiographic scores (Fig 4). Fifteen dogs were excluded because they either did not meet inclusion criteria in echocardiography (n = 5), had thoracic spine or vertebral deformities that considerably influenced radiographic scores (n = 7), or were poorly positioned or showed a distinct lack of cooperation (n = 3). In two dogs, left lateral images were insufficient due to poor positioning, so that the VHS LL could only be measured in a total of 30 dogs. On two of the 32 images in right lateral recumbency, measurements of VLAS and RLAD were not feasible due to deficient detectability of reference points by all observers. This led to a total of 30 measurements for VLAS and RLAD, respectively.

Of the 32 pugs eligible for radiographic measurements, 26 subjects were included for comparison of radiographic scores between BOAS- and BOAS+ pugs. Six dogs showed a lack of cooperation on the treadmill and thus could not receive a complete BOAS functional grading (lack of post-exercise evaluation).

Demographic data of all 32 pugs included in the radiographic measurements can be found in Table 2. Regarding demographic variables, all of these except age were normally distributed. Body weight was significantly higher in males than in females (p < 0.005). No significant differences of BW, BCS, and age between BOAS groups were detected. On examining radiographic measurements, all parameters were normally distributed.

**Table 2:** Demographic data of 32 pugs included in radiographic measurements.

N = 32	mean/median	SD/IQR	range (min—max)	
age* [years]	4.8	2.7–6.5	2–10.5	
body weight [kg]	8.9	1.2	6.6–11.6	
BCS	5.3	0.7	4–7	
LA:Ao	1.32	0.16	0.97–1.62	
female (n/i)	16 (8/8)			
male (n/i)	16 (3/13)			
BCS group (1-5/6-9)	21/11			
BOAS (N = 26)	BOAS- (N = 11) / BOAS+ (N = 15)			

Abbreviations: BCS, body condition score; BOAS, functional grading of Brachycephalic Obstructive Airway Syndrome; i, intact; IQR, interquartile range; kg, kilogram; LA:Ao, left-atrial-to-aortic-root diameter; max, maximum; min, minimum; N, number of subjects; n, neutered; SD, standard deviation.

Note: BOAS functional grading system according to Liu et al. 2015, modified to present study design. Median and IQR are stated for non-normally distributed variables, marked with an asterisk (*).

### Reference values for VHS, VLAS and RLAD

The 95% prediction interval and 90% CI for each reference limit were generated for VHS RL, VHS LL, VLAS, and RLAD from measurements of the most experienced examiner (observer 1) (Table 3). Significant differences were found for VHS RL and LL measured by observer 1 (p = 0.0002).

**Table 3:** Mean, median, SD, RI (95% prediction interval) and 90% CI for VHS, VLAS and RLAD.

	N	mean [v]	median [v]	SD	RI	(90% CI of RI)
**VHS RL**	32	11.25	11.2	± 0.62	10.1–12.8	(10.1–10.5)—(11.9–12.8)
**VHS LL**	30	11.01	11	± 0.70	9.4–12.6	(9.4–10.3)—(11.8–12.6)
**VLAS**	30	1.96	2.0	± 0.38	1.1–2.8	(1.1–1.6)—(2.3–2.8)
**RLAD**	30	1.59	1.6	± 0.34	0.7–2.4	(0.7–1.3)—(1.9–2.4)

Abbreviations: CI, confidence interval; N, number of subjects; LL, left lateral; RI, reference interval; RL, right lateral; RLAD, radiographic left atrial dimension; SD, standard deviation; v, thoracic vertebral unit; VHS, vertebral heart score; VLAS, vertebral left atrial size.

### Differences of radiographic scores regarding BOAS and other independent variables

The quantitative radiographic measurements VHS, VLAS and RLAD showed no significant differences between clinically non-affected and affected pugs (BOAS-/BOAS+) among all observers (for details, see supporting information; S1 Table). Regarding independent variables, the following observations were made: VHS, VLAS, and RLAD showed no significant differences between males and females, except for VHS RL in observer 2 where VHS was larger in males (p = 0.0349). The correlation of BW with radiographic scores was only significant for VLAS in observer 2 (*r* = 0.45, p = 0.0134). Significant differences between BCS groups could be detected for VHS LL in observers 2 and 3 (p = 0.0193 and p = 0.0272, respectively) and RLAD in observer 2 (p = 0.0369).

### Correlation of VLAS and RLAD to LA:Ao and VHS

On examining the correlation of radiographic scores to LA:Ao, weak correlations were detected for VLAS in all observers, being statistically significant in observer 2 (*r* = 0.39; p = 0.0332). VHS and RLAD showed no significant correlation to LA:Ao. Regarding VHS, the correlation of VLAS to VHS was significant in all observers (observer 1: *r* = 0.62, p = 0.0003; observer 2: *r* = 0.43, p = 0.0165; observer 3: *r* = 0.52, p = 0.0035). For RLAD, weak correlation to VHS could be detected, with the correlation coefficient being statistically significant for the measurements of observer 3 (*r* = 0.40; p = 0.0271).

### Differences between observers and intra- and interobserver agreement

Significant differences between radiographic scores among observers were detected for all radiographic scores. Mean, median, SD, and range of radiographic scores and significant differences between observers can be found in supplementary data (S3 Table).

Intraclass correlation coefficient for intraobserver agreement was almost perfect for VHS and VLAS (0.98 and 0.91, respectively) and moderate for RLAD (0.6; see Table 4). For interobserver agreement, ICC showed almost perfect agreement for VHS RL and LL (0.89 and 0.91), moderate agreement could be observed for VLAS (0.49), whereas RLAD showed fair agreement (0.22; for details, see Table 5).

**Table 4:** Intraobserver intraclass correlation coefficient (ICC) and 95% CI for VHS RL, VLAS, and RLAD.

	VHS RL	VLAS	RLAD
ICC	0.98	0.91	0.6
(95% CI)	0.92–0.99	0.6–0.98	0.18–0.87

Abbreviations: CI, confidence interval; RLAD, radiographic left atrial dimension; VHS RL, vertebral heart score right lateral recumbency; VLAS, vertebral left atrial size.

**Table 5:** Interobserver intraclass correlation coefficient (ICC) and 95% CI for VHS RL, VHS LL, VLAS, and RLAD.

	VHS RL	VHS LL	VLAS	RLAD
ICC	0.89	0.91	0.49	0.22
(95% CI)	0.79–0.94	0.82–0.97	0.27–0.7	0.07–0.52

Abbreviations: CI, confidence interval; RLAD, radiographic left atrial dimension; VHS RL/LL, vertebral heart score right lateral/left lateral recumbency; VLAS, vertebral left atrial size.

### Thoracic cavity and spine deformities

**Fig 5:**
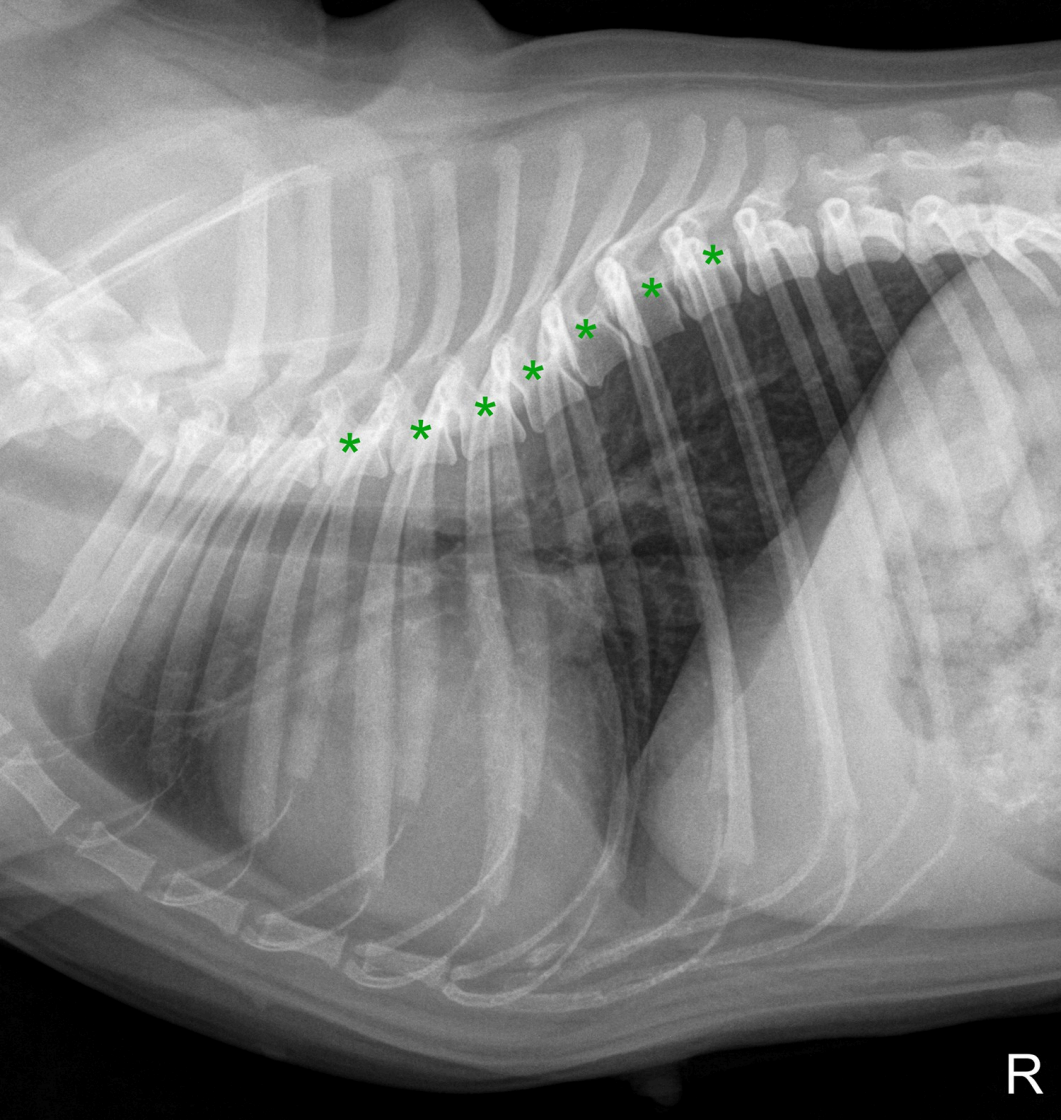
Right laterolateral thoracic radiograph of a five-year-old female pug showing irregularly shaped and trapezoid thoracic vertebrae throughout the thoracic spine (marked with asterisks), leading to exclusion from radiographic measurements.

**Fig 6:**
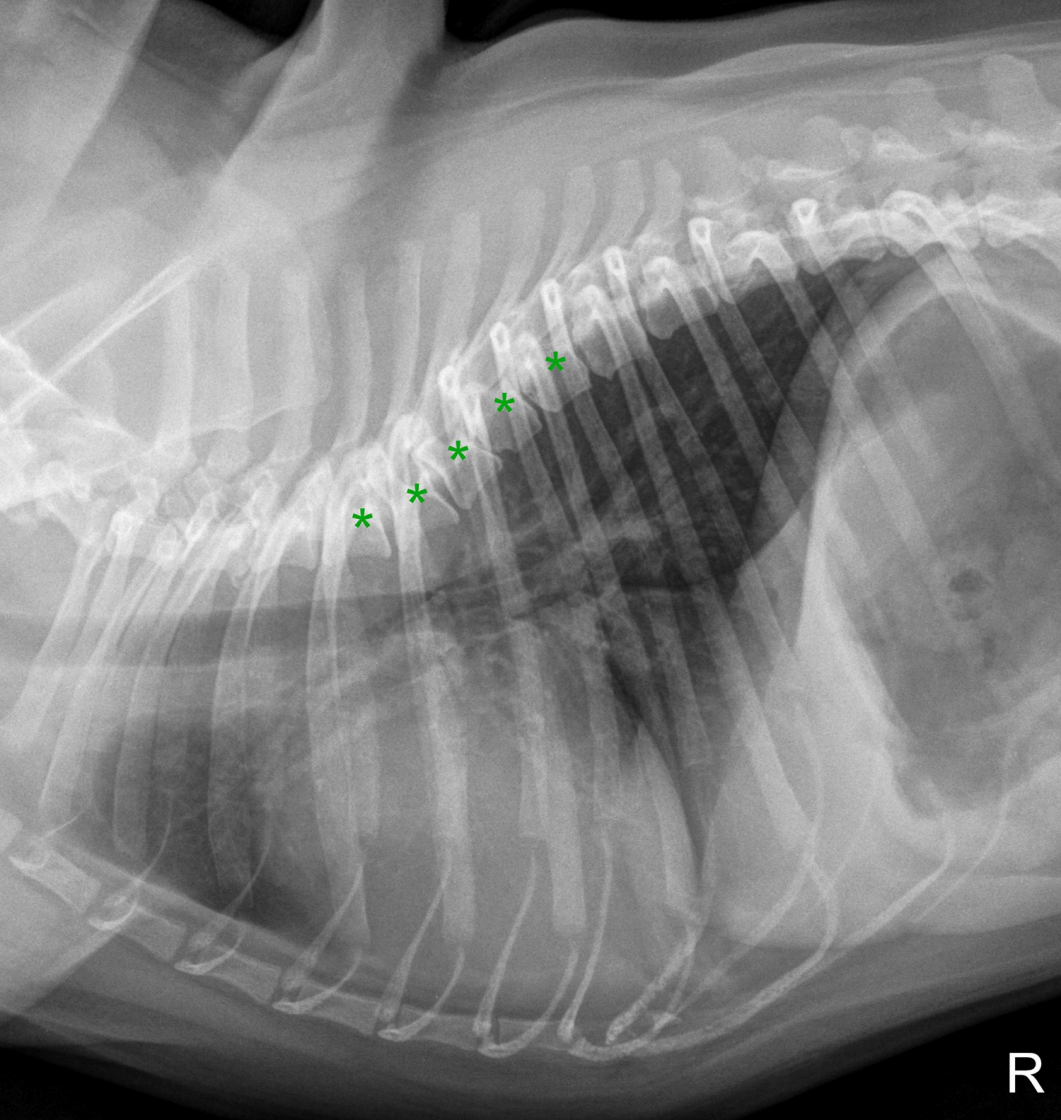
Right laterolateral thoracic radiograph of a four-year-old male pug with misshaped vertebral bodies from T4 to T8 (marked with asterisks), considerably influencing radiographic vertebral scores.

Of all 47 radiographically examined pugs, 18 (38.3%) pugs showed at least one abnormality of the thoracic cavity or spine. The most common finding was thoracic vertebral deformities in 12 pugs (25.5%). Nine dogs (19.1%) had irregularly shaped vertebrae, two (4.3%) had a ventrally wedge-shaped vertebra, and one (2.1%) had a dorsal hemivertebra. Six pugs (12.8%) presented an alteration of vertebral angulation (four pugs with focal kyphosis, one with focal lordosis, and one with general scoliosis). Focal kyphosis was directly associated with a specific vertebral malformation in two cases (one dorsal hemivertebra and one ventral wedge-shaped vertebra). In the other two cases and in the case of focal lordosis, irregular shapes of thoracic vertebral bodies were found to be the cause (Figs 5 and 6). Pectus excavatum was observed in one dog, PC in four dogs. Further, pugs showed a varying number of sternebrae and in 17 subjects (36.2%), no xyphoid could be detected. Spondylosis deformans was observed in 10 pugs (21.3%).

## Discussion

The purpose of this study was to determine values for VHS, VLAS, and RLAD in pugs without evidence of cardiac disease and to examine influences of BOAS and other variables (gender, BCS, BW). Furthermore, correlation of the left atrial scores to VHS and echocardiographic value LA:Ao were tested, intra- and interobserver agreement examined, and occurrences of thoracic cavity and spine deformities reported.

The present study proposes reference values for VHS RL (11.25v; RI, 10.1–12.8), VHS LL (11.01v; RI, 9.4–12.6), VLAS (1.96v; RI, 1.1v-2.8v), and RLAD (1.59v; RI, 0.7v-2.4v) in right lateral thoracic radiographs for pugs, adding to the growing pool of breed-specific radiographic reference values [17, 18, 22, 23, 48]. The calculations of reference values were based on the measurements of the most experienced observer (KM). This seemed the most reasonable approach to obtain high-quality measurements, as there were significant differences between the different observers, presumably due to varying levels of experience.

When considering the different scores, VHS was the score with the least interobserver differences: mean VHS measured by the observers merely differed between 0.06 to 0.18v and ICC was almost perfect for intra- and interreader agreement. These results are in agreement with findings of previous studies confirming VHS to be a well-established, reproducible radiographic score for measuring the size of the cardiac silhouette [16, 49]. Significant differences were found for VLAS and RLAD among the observers and additionally interobserver agreement turned out to be moderate for VLAS and only fair for RLAD. This is consistent with findings of a previous study examining interobserver variability for six observers for the evaluation of left atrial size in dogs of various breeds [37]. In the aforementioned study of Bagardi et al. 2021, significant differences in VHS but also RLAD and VLAS were found among observers, as well as among different levels of expertise and specialization. They further reported that when determining RLAD, identification of the dorsal margin of the left atrium may be impeded due to superimposition of the bronchial tree or pulmonary veins [37]. In the present study, all observers reported that decision-making for reference points was the most difficult for RLAD, and the two observers with less experience more frequently had difficulty identifying the dorsal reference point. This is reflected in the moderate intraobserver agreement for RLAD. It may be the main cause of differences in RLAD measurements among observers in the present study. It would further support the assumption that discrepancies between measurements of the observers mainly result from a different level of training, as presumed in the above-mentioned study [37]. This may additionally be affected by an even greater difficulty in finding the dorsal reference point in pugs because of a more pronounced overlap of bronchial structures and pulmonary veins due to chest conformation. Thus, measurement of VLAS and even more RLAD in pugs seem to be less reproducible and more observer-dependent than stated previously for dogs of other breeds [5, 8]. Especially clinicians lacking a high level of experience in radiographic evaluation of cardiac size should be cautious when quantitatively determining left atrial size. They may prefer VLAS in pugs, as it seems to be more reproducible and easier to measure than RLAD.

The potential for VLAS being more applicable than RLAD in pugs can be seen in the significant correlation to VHS in all observers, whereas RLAD merely weakly correlated to VHS, with only one observer (3) showing significant correlation. In previous studies, significant correlation to VHS has been observed in both VLAS and RLAD [8, 48–50].

Vertebral left atrial size is a good indicator for LAE in dogs with MMVD [13, 51] and was shown to be significantly more accurate than VHS in predicting echocardiographic LAE in dogs with cardiovascular disease [2]. Nevertheless, VLAS is more observer-dependent than VHS in pugs, and it should be considered that VHS may ultimately be more practical in pugs for determining cardiac dimensions in radiographs. However, the current study did not examine dogs with known cardiac disease and echocardiographic LAE, as it did not aim for a comparison to a diseased group, so this assumption cannot be definitively confirmed. Additionally, in the present study, only a weak correlation of VLAS to LA:Ao was found. Lack of a distinct correlation between radiographic and echocardiographic measurements in pugs may result from the circumstance that this study population consisted of dogs without cardiac disease and LAE. Thus, a narrow range of measurements may have impaired the possibility to detect a better correlation between VLAS and the echocardiographic left atrial size.

When comparing the breed-specific reference values with interbreed values, the established reference values for VLAS in pugs (2.0v) differed significantly compared to the interbreed values introduced by Malcolm et al. (2.1v, p = 0.0433) [5] and those of Chihuahuas (1.8v, p = 0.0303) [23] and Cavalier King Charles Spaniels (CKCS) (1.79v, p = 0.0220) [22]. On the other hand, VLAS did not differ significantly from interbreed references values (1.9v) [50] and values for Malteses (2.0v) [21]. Moreover, RLAD in pugs (1.59v) was significantly higher compared to the introductory study based on dogs of various breeds (1.41v, p = 0.0075) [8]. Likewise, RLAD was higher in pugs compared to CKCS (1.2v, p < 0.0001) [22]. Significant deviations from general reference values generated from various breeds should be kept in mind, and differences to both interbreed as well as to breed-specific studies underline the utility of references ranges for individual breeds.

Clinical grading of BOAS did not have significant influence on either VHS or radiographic left atrial measurements VLAS and RLAD. This suggests that radiographic scores can be used in pugs regardless of their clinical BOAS status. There is only one earlier study that investigated VHS measurements in relation to clinical signs of a breed-related upper airway disease in the Norwich Terrier [18]. No significant differences between the groups with and without respiratory disease were found in that study either.

The effect of BCS on VLAS and RLAD was investigated for the second time in the present study and no significant differences could be detected except for RLAD in observer 2. This is similar to findings of a previous study in CKCS [22]. For VHS, significantly higher values of dogs with a high BCS were detected for VHS LL in two observers (2 and 3), and a p value of 0.0527 in observer 1. As no differences between BCS groups were detected in VHS RL, this may have resulted from a greater difficulty in visually assessing fat opacity in left lateral recumbency due to a change of position of the heart in left lateral view. In the study by Avner and Kirberger (2005), it was postulated that a higher obesity grading, especially in small dogs, may lead to a greater movement of the cardiac apex in left lateral recumbency images [52]. This may explain higher VHS LL in the present study. Particularly in pugs, the examiner should consider changes in the cardiac silhouette in left lateral recumbency and pay special attention to limit the inclusion of fat when determining the most distal aspect of the cardiac silhouette.

Assessment of thoracic cavity and spine of all 47 dogs examined in the present study showed that there was a high occurrence of malformations in pugs. The present study population showed different abnormalities and prevalences than reported in previous studies [35, 53]. Even though a classification scheme has been proposed, particularly for brachycephalic “screw-tailed” breeds [33], nine of twelve pugs showed thoracic vertebral deformities in the present study and could not be classified by this specification. They did not show typical vertebral body malformation or segmentation defects, but rather irregularly shaped vertebrae which could be described more accurately as a generalized occurrence of misshaped, sometimes trapezoid vertebral bodies throughout the thoracic spine (see Figs 5 and 6). This variety in shape and vertebral body length even caused kyphosis and lordosis in three pugs. It further led to the exclusion from measurements in four cases, as radiographic scores would have been considerably altered. Influence of vertebral deformities in VHS has been observed for the Bulldog and the Boston Terrier, with VHS being significantly larger in dogs with abnormal vertebrae [20]. Therefore, special attention should be given to the shape of thoracic vertebrae when determining radiographic scores in pugs, as abnormalities of the thoracic spine may lead to false results. In cases where inexperienced examiners evaluate radiographs, they should be aware that the radiographic measurements can be notedly influenced by vertebral deformities in pugs, even if they are non-classifiable, which may reduce their clinical validity.

One limitation in the present study is the sample size. According to recent guidelines, a subject sample of > 120 is preferable for creating valid reference values in veterinary medicine [45]. However, the number of subject samples of previous veterinary studies has often been less than the recommended amount, and the amount of pugs included for measurements in the present study is comparable to those of others investigating breed-specific ranges for VHS, VLAS, and RLAD [14–18, 20, 22, 54]. Nevertheless, measurements should still be interpreted with caution. Furthermore, vertebral malformations were subjectively assessed in the present study and many dogs were presented with non-classifiable deformities.

## Conclusions

In conclusion, breed-specific reference values for VHS RL, VHS LL, VLAS and RLAD were established in this study. The VLAS of pugs were statistically different to some studies but in general showed quite similar values compared to existing studies concerning interbreed and breed-specific reference values. In contrast, RLAD was significantly higher in pugs compared to interbreed values. The radiographic scores VHS, VLAS, and RLAD were not significantly influenced by the presence of clinical signs of BOAS. Both VLAS and RLAD have to be used with caution, as they have a moderate to fair interobserver agreement. The common occurrence of spinal malformations in pugs might alter the values of radiographic scores referring to vertebral body length, and this should be kept in mind if applied in such patients.

## Supporting information

S1 Table BOAS functional grading system based on publication of Liu et al. 2015. (DOCX)

S2 Table Measurements of each radiographic score by each observer, subdivided into BOAS non-affected and affected group (BOAS-/BOAS+). (DOCX)

S3 Table Measurements of radiographic scores of each observer and significant differences between observers. (DOCX)

S1 File Dataset. (XLSX)
