# Supplementary material for: Breed-specific values for vertebral heart score (VHS), vertebral left atrial size (VLAS), and radiographic left atrial dimension (RLAD) in pugs without cardiac disease, and their relationship to Brachycephalic Obstructive Airway Syndrome (BOAS)
Source: PLoS One. 2022 Sep 2;17(9):e0274085. doi: 10.1371/journal.pone.0274085 (PMC9439199; doi:10.1371/journal.pone.0274085)
Supplement: S1 Table — (DOCX) [file pone.0274085.s001.docx]

**S1 Table. BOAS functional grading system based on publication of Liu et al 2015.**

|  |  | Respiratory noise^a^ | Inspiratory effort^b^ | Dyspnea/Cyanosis/Syncope^c^ |
| --- | --- | --- | --- | --- |
| Grade 0 | Pre-ET | Not audible | Not present | Not present |
|  | Post-ET | Not audible | Not present | Not present |
| Grade 1 | Pre-ET | Not audible or mild | Not present | Not present |
|  | Post-ET | Mild | Not present to mild | Not present |
| Grade 2 | Pre-ET | Mild to moderate | Mild to moderate | Not present |
|  | Post-ET | Moderate to severe | Moderate to severe | Mild dyspnea; cyanosis or syncope not present |
| Grade 3 | Pre-ET | Moderate to severe | Moderate to severe | Moderate to severe dyspnea; may or may not present cyanosis. Inability to exercise. |
|  | Post-ET | Severe |  | Severe dyspnea; may or may not present cyanosis or syncope. |

The clinical grading was based on respiratory signs before (pre-ET) and after an exercise test (post-ET), modified to match the present study design (Liu et al. 2015): Dogs underwent a submaximal ET in an individual trotting pace on a treadmill and post-ET grading was conducted at the end of the ET, after 15 minutes of exercise. Grades 0 and 1 were summarized as a clinically non-affected (BOAS-) group and Grades 2 and 3 as a clinically affected (BOAS+) group (Liu et al. 2015).

^a^Respiratory noise was diagnosed by pharyngolaryngeal and thoracic auscultation. Mild: only audible under auscultation; moderate: intermittent audible noise that can be heard without stethoscope; severe: constant audible noise that can be heard without stethoscope.

^b^An abnormal respiratory cycle characterized by evidence of increased effort to inhale the air with the use of diaphragm and/or accessary muscles of respiration and/or nasal flaring with an increase in breathing rate. Mild: regular breathing patterns with minimal use of diaphragm; moderate: evidence of use of diaphragm and accessary muscles of respiration; severe: marked movement of diaphragm and accessary muscles of respiration.

^c^Dogs that have had episodes of syncope and/or cyanosis as documented by owner's report are classified as Grade 3 without ETT. Mild dyspnea: shows sign of discomfort; moderate dyspnea: irregular breathing, signs of discomfort; severe dyspnea: irregular breathing with signs of breathing discomfort and difficulty in breathing.
