# Supplementary material for: Breed-specific values for vertebral heart score (VHS), vertebral left atrial size (VLAS), and radiographic left atrial dimension (RLAD) in pugs without cardiac disease, and their relationship to Brachycephalic Obstructive Airway Syndrome (BOAS)
Source: PLoS One. 2022 Sep 2;17(9):e0274085. doi: 10.1371/journal.pone.0274085 (PMC9439199; doi:10.1371/journal.pone.0274085)
Supplement: S2 Table — (DOCX) [file pone.0274085.s002.docx]

**S2 Table. Measurements of each radiographic score by each observer, subdivided into BOAS non-affected and affected group (BOAS-/BOAS+).**

| **N = 26** |  |  | **BOAS-** |  | **BOAS+** | **p value** |
| --- | --- | --- | --- | --- | --- | --- |
| observer 1 |  | N | mean ± SD | N | mean ± SD |  |
|  | **VHS RL** | 11 | 11.3 ± 0.68 | 15 | 11.2 ± 0.57 | 0.8352 |
|  | **VHS LL** | 11 | 11.1 ± 0.61 | 13 | 11.1 ± 0.61 | 0.9874 |
|  | **VLAS** | 11 | 2.03 ± 0.48 | 13 | 1.89 ± 0.37 | 0.5035 |
|  | **RLAD** | 10 | 1.55 ± 0.44 | 15 | 1.64 ± 0.31 | 0.5413 |
| observer 2 |  |  |  |  |  |  |
|  | **VHS RL** | 11 | 11.4 ± 0.66 | 15 | 11.4 ± 0.63 | 0.8555 |
|  | **VHS LL** | 11 | 11.3 ± 0.80 | 13 | 11.4 ± 0.58 | 0.7495 |
|  | **VLAS** | 11 | 1.8 ± 0.27 | 13 | 1.78 ± 0.39 | 0.7804 |
|  | **RLAD** | 10 | 1.34 ± 0.28 | 15 | 1.39 ± 0.45 | 0.8610 |
| observer 3 |  |  |  |  |  |  |
|  | **VHS RL** | 11 | 11.45 ± 0.73 | 15 | 11.4 ± 0.63 | 0.9384 |
|  | **VHS LL** | 11 | 11.25 ± 0.74 | 13 | 11.4 ± 0.69 | 0.4500 |
|  | **VLAS** | 11 | 2.14 ± 0.35 | 13 | 2.05 ± 0.51 | 0.6339 |
|  | **RLAD** | 10 | 1.85 ± 0.34 | 15 | 1.91 ± 0.38 | 0.9071 |

Abbreviations: BOAS, Brachycephalic Obstructive Airway Syndrome; N, number of subjects; RLAD, radiographic left atrial dimension; SD, standard deviation; VHS RL/LL, vertebral heart score right lateral/left lateral recumbency; VLAS, vertebral left atrial size
