# Supplementary material for: Breed-specific values for vertebral heart score (VHS), vertebral left atrial size (VLAS), and radiographic left atrial dimension (RLAD) in pugs without cardiac disease, and their relationship to Brachycephalic Obstructive Airway Syndrome (BOAS)
Source: PLoS One. 2022 Sep 2;17(9):e0274085. doi: 10.1371/journal.pone.0274085 (PMC9439199; doi:10.1371/journal.pone.0274085)
Supplement: S3 Table — (DOCX) [file pone.0274085.s003.docx]

**S3 Table. Measurements of radiographic scores of each observer and significant differences between observers.**

|  | **N** | **mean ± SD [v]** | **median [v]** | **range (min - max)** | **p value** |
| --- | --- | --- | --- | --- | --- |
| **VHS RL** | 32 |  |  |  | 0.0069^a^ |
| observer 1 |  | 11.25 ± 0.62 | 11.2 | 10.1-12.8 |  |
| observer 2 |  | 11.43 ± 0.66 | 11.3 | 10-12.7 |  |
| observer 3 |  | 11.37 ± 0.66 | 11.45 | 10-12.9 |  |
|  |  |  |  |  |  |
| **VHS LL** | 30 |  |  |  | < 0.0001^b^ |
| observer 1 |  | 11.01 ± 0.7 | 11 | 9.4-12.6 |  |
| observer 2 |  | 11.24 ± 0.71 | 11.15 | 10.0-12.9 |  |
| observer 3 |  | 11.21 ± 0.71 | 11 | 10-12.7 |  |
|  |  |  |  |  |  |
| **RLAD** | 30 |  |  |  | < 0.0001^c^ |
| observer 1 |  | 1.59 ± 0.34 | 1.6 | 0.7-2.4 |  |
| observer 2 |  | 1.87 ± 0.35 | 1.4 | 0.6-2.0 |  |
| observer 3 |  | 1.36 ± 0.37 | 1.9 | 1.2-2.8 |  |
|  |  |  |  |  |  |
| **VLAS** | 30 |  |  |  | 0.0002^d^ |
| observer 1 |  | 1.96 ± 0.38 | 2 | 1.1-2.8 |  |
| observer 2 |  | 2.07 ± 0.41 | 1.8 | 1.1-2.3 |  |
| observer 3 |  | 1.78 ± 0.33 | 2 | 0.8-2.8 |  |

a: VHS RL differs significantly among observers in Friedman test (p = 0.0069)

b: VHS LL differs significantly among observers in Friedman test (p < 0.0001)

c: RLAD differs significantly among observers in Friedman test (p < 0.0001)

d: VLAS differs significantly among observers in Friedman test (p = 0.0002)

Abbreviations: N, number of subjects; RLAD, radiographic left atrial dimension; SD, standard deviation; v, thoracic vertebral unit, VHS RL/LL, vertebral heart score right lateral/left lateral recumbency; VLAS, vertebral left atrial size
